# Regulatory Mechanisms of Anthocyanin Biosynthesis in Apple and Pear

**DOI:** 10.3390/ijms22168441

**Published:** 2021-08-06

**Authors:** Huimin Liu, Zijin Liu, Yu Wu, Lamei Zheng, Genfa Zhang

**Affiliations:** 1 Beijing Key Laboratory of Gene Resource and Molecular Development, College of Life Sciences, Beijing Normal University, Beijing 100875, China; Huimin_Liu521@163.com (H.L.); liuzijin1012@163.com (Z.L.); 18810936228@163.com (Y.W.); zhengIm100@163.com (L.Z.); 2 State Key Laboratory of Crop Stress Biology for Arid Areas and College of Agronomy, Northwest A&F University, Yangling, Xianyang 712100, China

**Keywords:** anthocyanins, phytohormones, light and temperature, transcription factors, molecular mechanisms

## Abstract

Anthocyanins contribute to the quality and flavour of fruits. They are produced through the phenylpropanoid pathway, which is regulated by specific key genes that have been identified in many species. The dominant anthocyanin forms are reversibly transformed at different pH states, thus forming different colours in aqueous solutions. In plants, anthocyanins are controlled by specific factors of the biosynthetic pathway: light, temperature, phytohormones and transcription factors. Although great progress in research on anthocyanin structures and the regulation of anthocyanin biosynthesis has been made, the molecular regulatory mechanisms of anthocyanin biosynthesis in different plants remain less clear. In addition, the co-regulation of anthocyanin biosynthesis is poorly understood. In this review, we summarise previous findings on anthocyanin biosynthesis, including the biochemical and biological features of anthocyanins; differences in anthocyanin biosynthesis among fruit species, i.e., apple, red pear, and the model plant *Arabidopsis thaliana*; and the developmental and environmental regulation of anthocyanin accumulation. This review reveals the molecular mechanisms underlying anthocyanin biosynthesis in different plant species and provides valuable information for the development of anthocyanin-rich red-skinned and red-fleshed apple and pear varieties.

## 1. Introduction

The accumulation of different pigmentations affects the growth, development and reproduction of plants under various conditions, which, similar to the diversification of coat hair colour in mammals, is appropriate for different environments [1]. Anthocyanins are water-soluble flavonoid pigments widely distributed in the petals, fruits, stems and leaves of plants. [2,3,4]. In addition, anthocyanins are natural antioxidants that can strongly scavenge free radicals and reactive oxygen species (ROS) [5]. Anthocyanins have a pivotal role in the rendering of red, purple and blue colours of plant tissues and organs, and contribute to the dispersal of pollen and seeds by attracting animals such as bees and birds [6,7]. Moreover, anthocyanins are also important indicators of fruit ripening and influence the quality of fruits and their derived products (e.g., wine and fruit juices), which affects the purchasing behaviour of consumers to a certain extent [8].

In the past few decades, great progress in the understanding of anthocyanin metabolism in model plant species has been made, especially with respect to the biosynthetic pathways of these compounds. In plants, anthocyanin biosynthesis is affected by various biotic and abiotic stresses, including UV irradiation, insect attack, drought, and low temperature [9,10,11]. Additionally, the accumulation of anthocyanins is also determined by enzyme-coding structural genes, transcription factors, plant hormones and microRNAs [12,13,14,15].

Anthocyanins participate in the formation of fruit quality, affecting taste, colour and lustre, whilst even affecting human health. The biosynthesis of anthocyanins in fruit crops has attracted large amounts of attention from academics. With the development of molecular biology and bioinformatics, an increasing number of studies have revealed that there are obvious differences in the regulatory mechanisms of anthocyanin biosynthesis between different plant species. Many key players in the complex regulatory network remain to be identified. Therefore, this article aims to provide a detailed overview of the known regulatory mechanisms of anthocyanin biosynthesis in apples and pears and provide a theoretical foundation for the genetic improvement and breeding of crops, fruits and ornamental plant species [16].

## 2. Basic Information of Anthocyanins

### 2.1. Classification and Chemical Structure of Anthocyanins

**Figure 1:**
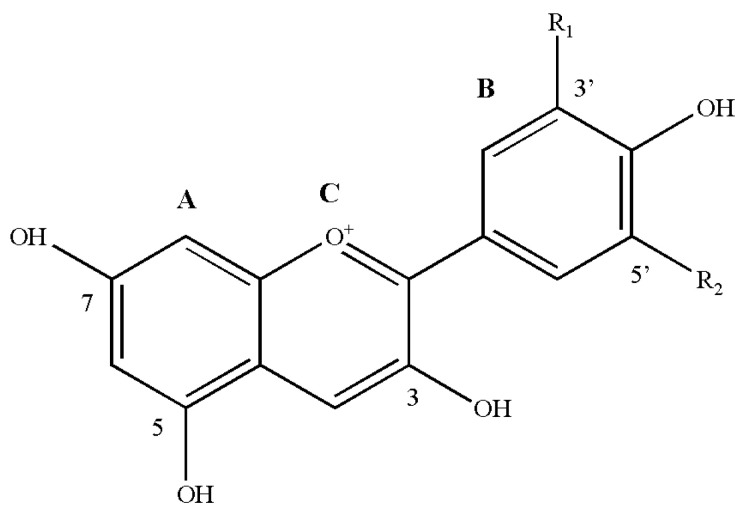
Basic chemical structures of anthocyanidins. A, C6; B, C6; C, C3, anthocyanins consisting of α-phenylbenzopyran cations are mainly composed of C6-C3-C6 carbon skeleton structures. R1 and R2, structural groups at different positions (3′ and 5′) on the B ring. Detailed information on R1 and R2 substitutions is listed in Table 1.

Anthocyanins are important secondary metabolites belonging to the flavonoids (polyphenols). At present, more than twenty kinds of anthocyanins have been identified in nature, and approximately 90% of more than 550 kinds of anthocyanins found in nature are derived from the six most common anthocyanins: cyanidin (Cy), peonidin (Pn), pelargonidin (Pg), malvidin (Mv), delphinidin (Dp) and petunidin (Pt). Anthocyanins exist alongside a variety of monosaccharides, including glucose, rhamnose, galactose and xylose, and disaccharides consisting of rhamnose, gentian disaccharide and sophora disaccharide to form glycosides. Additionally, anthocyanins consist of α-phenylbenzopyran cations and are mainly composed of C6(A)-C3(C)-C6(B) carbon skeleton structures (Figure 1). At the A and C rings of these six anthocyanins, the 3, 5 and 7 positions connect to the hydroxyl group, while at the B ring, the hydroxyl group is connected to position 4′. Whether positions 3′ and 5′ have hydroxyl groups and are methoxylated is considered the standard for distinguishing the six anthocyanins. Due to the methylation and hydroxylation modifications at different positions of the ring, different colours of anthocyanins are formed (Table 1).

### 2.2. The Influence of pH on Anthocyanin Chemical Structure

**Figure 2:**
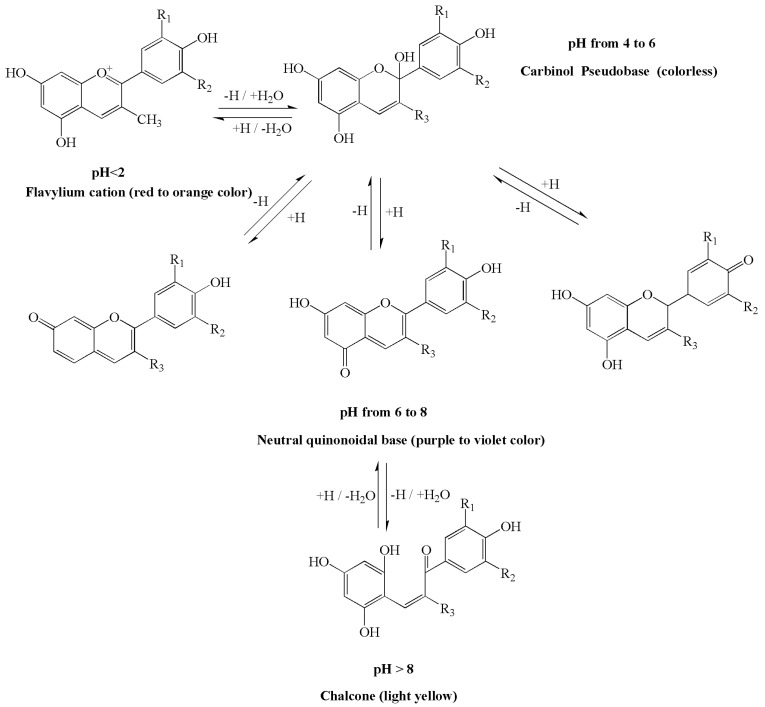
Scheme of the pH-dependent structural equilibrium interconversion between the dominant anthocyanin forms. Four dominant anthocyanin forms, flavylium cation, carbinol pseudobase, neutral quinonoidal base and chalcone, can reversibly transform at different pH states and then produce different colours. pH < 2, the colour ranges from red to orange; 4 < pH < 6, shows mainly a colourless solution; 6 < pH < 8, the colours vary from purple to violet; pH > 8 a light yellow and colourless solution appears.

The corresponding four dominant anthocyanin forms, flavylium cations, carbinol pseudobases, neutral quinonoidal bases and chalcone, are reversibly transformed at different pH states (Figure 2), and so different colours will be produced concomitantly in aqueous solutions [17]. Under acidic conditions, three chemical equilibria, including acid-base equilibrium, hydration equilibrium and ring-chain tautomeric equilibrium, were found to coexist. When the pH < 2, anthocyanin exists in the form of red flavylium cations; as the pH increases, the flavylium cation rapidly continues to hydrate to produce a colourless carbinol pseudobase at a pH from 4 to 6. Under alkaline conditions, acid-base equilibrium becomes dominant, and proton transfer reactions swiftly occur to generate products that are unstable and that easily degrade into other products. As the solution pH value varies from 6 to 8, the colours vary from purple to violet. When the pH > 8, anthocyanins exist in the form of chalcone, and a colourless solution emerges. Under the same external conditions, the higher the pH, the faster the degradation rate of anthocyanins [18]. In addition, anthocyanins are unstable in nature, approximately 65% of anthocyanins are acylated, and glycosylation and methylation promote the polymorphism of anthocyanins. The presence of metal ions, oxidative degradation, vitamin C, sugars, temperature and light also affects the stability of the anthocyanin chemical structure [19,20].

## 3. Biosynthesis Pathway of Anthocyanins

**Figure 3:**
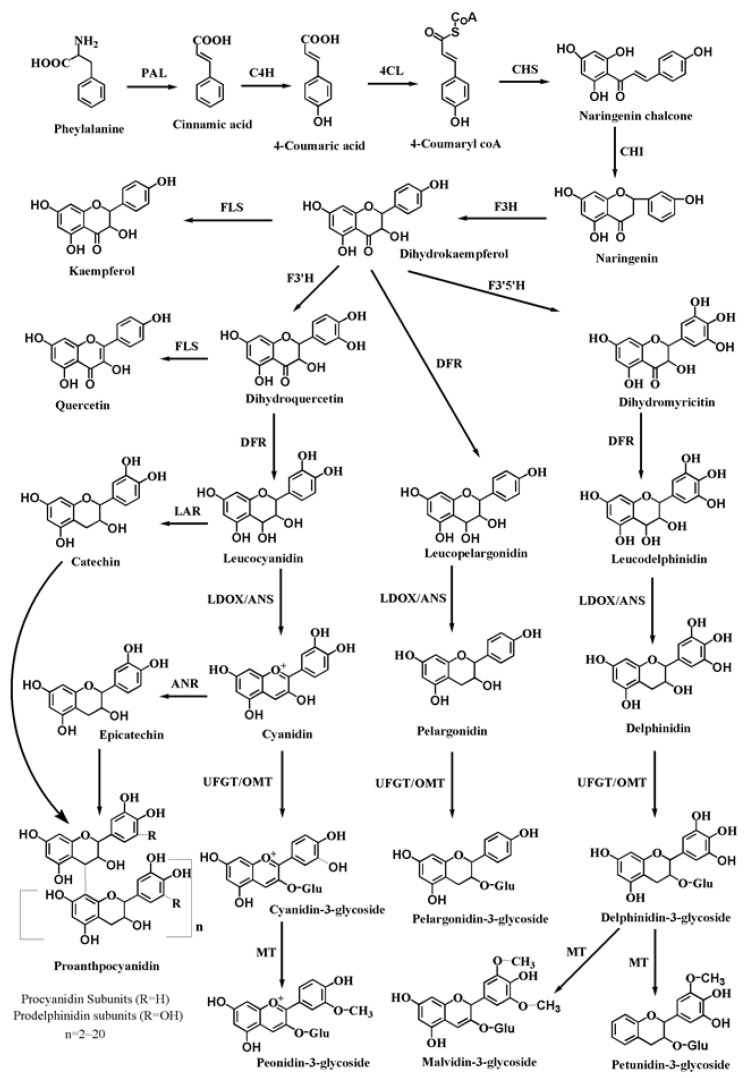
The biosynthetic pathway of anthocyanins/proanthocyanidins. PAL, phenylalanine ammonia lyase; C4H, cinnamate 4-hydroxylase; 4CL, 4-coumarate CoA ligase; CHS, chalcone synthase; CHI, chalcone isomerase; F3H, flavanone 3-hydroxylase; F3′H, flavonoid 3′ hydroxylase; F3′5′H, flavonoid 3′5′hydroxylase; FLS, flavonol synthase; DFR, dihydroflavonol 4-reductase; LAR, leucoanthocyanidin reductase; ANR, anthocyanidin reductase; ANS, anthocyanidin synthase; LDOX, leucoanthocyanidin dioxygenase; UFGT, UDP-galactose flavonoid 3-O-galactosyltransferase; OMT, O-methyl transferase; MT, methyltransferase.

Anthocyanins are synthesised from a common phenolic precursor through the phenylpropanoid pathway on the cytoplasmic face of the endoplasmic reticulum in plants. Stable anthocyanins formed by different modifications, such as glycosylation, methylation and acylation, are transported into the vacuole, where they accumulate. As one of the end-products of the flavonoid biosynthetic pathway, proanthocyanidins are synthesised from epicatechin, which is catalysed by anthocyanidin reductase (ANR) and subsequently transported and polymerised [21]. The correlative enzymes and central regulatory factors involved in the anthocyanin biosynthetic pathway are shown in schematic models (Figure 3). In the anthocyanin biosynthetic pathway, multiple enzyme-encoding structural genes are involved as follows: upstream structural genes, such as phenylalanine ammonialyase (*PAL*) and 4-coumarate–CoA ligase (*4CL*); early biosynthetic genes (EBGs), such as chalcone synthase (*CHS*), chalcone isomerase (*CHI*), flavanone 3-hydroxylase (*F3H*), flavonoid 3′-hydroxylase (*F3′H*), and flavonoid 3′5′-hydroxylase (*F3′5′H*); late biosynthetic genes (LBGs), such as dihydroflavonol 4-reductase (*DFR*), anthocyanidin synthase/leucoanthocyanidin dioxygenase (*ANS/LDOX*), and UDP-glucose flavonoid glucosyl transferase (*UFGT*); and modifying genes, such methyltransferase (*MT*), O-methyltransferase (*OMT*) and anthocyanin transferase (*AT*). In addition, four structural genes, transparent taste 10 (*tt10*), transparent taste 12 (*tt12*), transparent taste 13 (*tt13*) and transparent taste 19 (*tt19*), encode polyphenol oxidase (PPO), a secondary transport factor of the MATE (multidrug and toxic compound extrusion) family, H (+)-ATPase and glutathione S-transferase (GST), respectively. These proteins play significant roles in the modification, transport and oxidation of anthocyanidins [22,23,24].

The synthesis of anthocyanins in plants is controlled by regulatory genes. At present, the identified genes involved in the regulation of the anthocyanin biosynthetic pathway consist of the myeloblastosis family (MYB) TFs, the basic helix-loop-helix (bHLH) TFs and the tryptophan-aspartic acid repeat (WDR) TFs [25,26]. Generally, the members of these three families of regulatory factors mainly depend on the MYB-bHLH-WD40 (MBW) complex to exert their effects [27,28].

## 4. Regulatory Mechanism of Anthocyanin Biosynthesis

**Figure 4:**
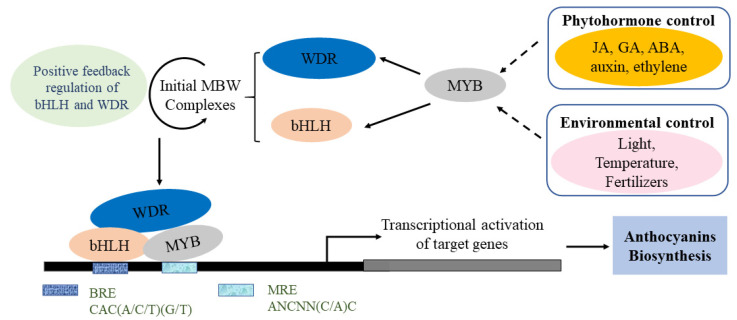
Simplified model of the regulatory mechanisms of anthocyanin biosynthesis. Developmental and environmental factors could induce MYB, which then activates WDR and bHLH to form the MBW complex. The MBW complex consists of MRE (MYB recognition elements) and BRE (bHLH recognition elements), which bind to the promoter of the target gene. The transcriptional activation of target genes promotes anthocyanin biosynthesis.

The biosynthesis of anthocyanins is affected by many factors, and therefore the regulatory mechanism is complicated in plants. To date, the main factors include environmental factors, phytohormones, transcription factors and epigenetic modification. A schematic of the regulatory mechanism of the activation of anthocyanin biosynthesis is summarised briefly (Figure 4). In plants, MYB TFs are characterised by the highly conserved MYB domain, with MYB proteins usually interacting with bHLH factors and WD-repeat proteins in order to regulate anthocyanin biosynthesis [29]. Internal factors such as plant hormones, and external factors such as light, temperature, fertility, sucrose and drought, can both highly affect the transcriptional activation of target genes and the biosynthesis, accumulation and transport of anthocyanins [30,31]. Anthocyanin metabolic pathways and related gene transcription in different plant species respond to changes under different conditions.

### 4.1. Light and Temperature

Light is essential for plant growth and development, and especially affects anthocyanin biosynthesis. After photostimulation, the photoreceptors activate a range of signal transduction pathways involving photoreactions and corresponding gene expression [11]. In this process, some central light signal elements, such as constitutive photomorphogenic 1 (COP1), suppressor of PYHA (SPA) and elongated hypocotyl 5 (HY5), are involved. In general, HY5 itself can target but not activate the promoters of anthocyanin biosynthesis-related genes [32,33]. Once HY5 accumulates in abundance, it will be degraded by COP1 because of the photoinhibition of COP1 activity [34,35]. Some BBX proteins interact with HY5 and further induce anthocyanin biosynthesis [36,37]. At present, most of the research on the regulation of anthocyanin biosynthesis by light focuses on apple and pear.

**Figure 5:**
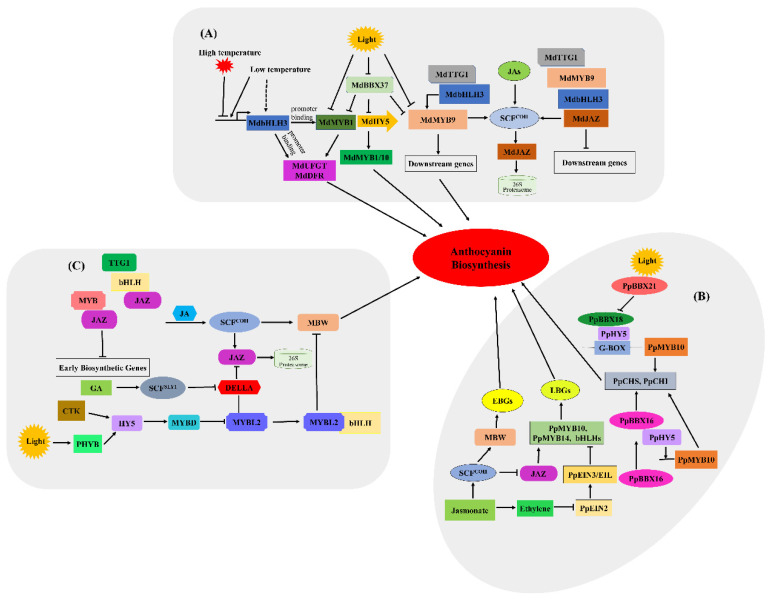
Simplified model for light, temperature, phytohormones and transcription factors involved in regulating anthocyanin biosynthesis [41]. (**A**) Light, temperature and transcription factors involved in regulating anthocyanin biosynthesis in red-fleshed apples. (**B**) Phytohormones and light are involved in regulating anthocyanin biosynthesis in red pears. (**C**) Light and phytohormone-induced regulation of anthocyanin biosynthesis in *A. thaliana*.

In apples (Figure 5A), light negatively regulates anthocyanin biosynthesis mainly by inhibiting the expression of *MdBBX37*. MdBBX37 belongs to the zinc finger transcription factor family, whose members contain at least one conserved B-box motif in the N-terminal region [38,39]. MdBBX37 reduces the expression level of *MdHY5* by directly targeting the promoter of *MdHY5*. Conversely, MdWRKY72 and MdWRKY11 transcription factors bind to the W-box cis-element of *MdHY5* to activate its regulation of anthocyanin biosynthesis [20,40]. Thus, the function of BBX proteins in response to light-induced anthocyanin accumulation requires the participation of HY5 [36,37]. Moreover, as a bZIP TF and positive regulator of light signalling, MdHY5 can enhance anthocyanin biosynthesis by directly activating the expression of *MdMYB1/10* [34]. However, MdBBX37 hinders the binding of MdMYB1 and MdMYB9 to their target genes and further reduces anthocyanin accumulation [41,42,43].

In red pears, PpBBX16, a positive regulator of light-induced anthocyanin accumulation, cannot combine directly with the promoter of either PpMYB10 or PpCHS, but can interact with PpHY5 physically to form the PpBBX16-PpHY5 complex (Figure 5B). PpHY5 directly binds to the *PpMYB10* promoter, but whether PpHY5 can activate gene expression has not been proven [44]. The PpBBX16-PpHY5 complex stimulates the promoter activity of *PpMYB10* and subsequently strongly enhances light-induced anthocyanin accumulation, and the overexpression of *PpBBX16* also promotes anthocyanin accumulation in the peel of pear fruits [45]. As a positive regulator, PpBBX18 directly interacts with PpHY5, and the heterodimer PpBBX18-pHY5 regulates anthocyanin accumulation by inducing *PpMYB10* transcription [46]; conversely, as a negative regulator, PpBBX21 can physically interact with PpBBX18 and PpHY5, repressing anthocyanin biosynthesis by hindering the formation of the PpHY5-PpBBX18 complex [44,47]. In addition, HY5 binds to the *MYBD* promoter directly in *A. thaliana*, which positively accelerates the expression of downstream genes in light- or cytokinin-stimulated signalling pathways [40,48,49].

A large number of studies have also revealed that ambient temperature is a key factor regulating the accumulation of anthocyanins. In general, high temperature represses while low temperature promotes the biosynthesis of anthocyanin [50,51,52]. It is likely that the COP1-HY5 module may also play an important role in this process. When the temperature is elevated, COP1 is imported to nucleus and further decreases the biosynthesis of anthocyanins by destabilising HY5 [53]. In contrast, cold temperature depletes COP1 from the nucleus, resulting in HY5 stabilisation and an increase in anthocyanin production [54]. Moreover, low temperature enhances the transcription of MdbHLH3 and promotes the activity of structural genes involved in anthocyanin biosynthesis by phosphorylating MdbHLH3 [31]. Detailed regulatory mechanisms of temperature on anthocyanin biosynthesis still need to be explored.

### 4.2. Phytohormones

Phytohormones are indispensable for the processes of plant growth and development, and known plant hormones, such as JA (jasmonate), ABA (abscisic acid), ethylene and auxin, could stimulate the ethylene response factor family (ERF) and be involved in the regulation of anthocyanin biosynthesis [55,56,57]. In red pear and *A. thaliana*, JAZ (jasmonate ZIM-domain) proteins are substrates of the SCF^COI1^ complex, which interferes with the transcriptional levels of WD/bHLH/MYB complexes by interacting directly with the C-terminus of both bHLH and MYB members, in turn repressing anthocyanin biosynthesis [58]. Moreover, JAZs act as negative regulatory factors in apples [59]. The direct interaction of MdJAZ with MdbHLH3 interferes with the recruitment of MdbHLH3 to the *MdMYB9* promoter, which represses the transcription of the MBW complexes and further decreases anthocyanin biosynthesis [44]. In addition, ABI5 promotes ABA-induced anthocyanin biosynthesis by regulating the MYB1-bHLH3 complex in apples [60]. Ethylene inhibits anthocyanin biosynthesis by downregulating the expression of R2R3-MYBs (including *PpMYB10* and *PpMYB114*) and LBGs (late biosynthetic genes) in red pears, and ethylene signal transduction occurs via ethylene response factors (ERFs) and EIN3/EILs (ethylene-insensitive 3, EIN3; EIN3-like, EIL) [57]. Usually, high levels of auxin can inhibit anthocyanin biosynthesis by suppressing regulatory genes and structural genes [45,61]. Cytokinin plays a positive role in photomorphogenesis in *Arabidopsis thaliana*, while GA signalling curbs anthocyanin biosynthesis via DELLA proteins [62,63]. DELLAs may also be essential for the function of gibberellic acids (GA) in regulating the biosynthesis of anthocyanins. In the presence of GA, DELLAs are polyubiquitinated and then degraded by the 26S proteasome [64].

### 4.3. Transcription Factors

The expression of structural genes during anthocyanin biosynthesis is directly controlled by the MYB-bHLH-WDR complex. R2R3-MYB transcription factors play a critical role in this regulatory pathway, which can directly regulate the expression of related genes and lead to tissue-specific anthocyanin accumulation [65,66]. BHLH transcription factors are indispensable for the activity of R2R3-MYBs, mainly by stabilising the MYB complex or promoting its transcription [29,67]. For example, some MYB transcription factors, such as MdMYB1, MdMYB9, MdMYB10 and MdMYB114, can promote apple fruit colouration by means of interaction with bHLH3 and WD40 [42,68]. In fleshy fruit skin, MdMYB1 is initially found in apple skin, and its product participates in photoinduction by activating the transcription activity of MdDFR and MdUFGT promoters. The transcript level of *MdMYB1* is positively correlated with the accumulation of anthocyanin and the expression of structural genes because the *MdDFR* and *MdUFGT* promoters include light-response elements, such as ACGTs and MRE or MRE-like sequences [43,69]. Members of the WD40 protein family have 4–10 random WD repeat domains, which consist of 40 amino acid sequences ending in tryptophan (W) and aspartic acid (D). MdTTG1 was the first WD40 protein isolated from apple, similar to AtTTG1 in Arabidopsis, MdTTG1 can interact with MdbHLH3 and MdMYB9 to control the expression of downstream structural genes [70]. Similarly, PpMYB10 and PpMYB114 also contribute to the colouration of red pears. However, some MYB TFs, such as Arabidopsis MYBL2, apple MYB16, peach MYB17-20, and strawberry MYB1, negatively regulate anthocyanin biosynthesis [71,72,73]. The interaction of MYBL2 with bHLH hinders the formation of the MBW complex, and MYBL2 competitively interacts with the MYB/bHLH and bHLH subunits. Once the expression level of *MYBL2* is reduced, the expression of early biosynthetic genes and the accumulation of anthocyanin increase (Figure 5C). Given that TFs from the same family have different functions in the regulation of anthocyanin biosynthesis, the functional elucidation of more TFs will be a fertile research area in the coming years.

### 4.4. Other Regulatory Factors

Demethylation and methylation of DNA also have important roles in regulating anthocyanin accumulation, and studies have shown that the DNA methylation inhibitor 5-azacytidine can induce red pigmentation in apple and peach fruits [74,75]. Coincidentally, in red-fleshed radish, DNA methylation of the RsMYB1 promoter inhibits anthocyanin biosynthesis [76]. In addition, microRNAs also play vital roles in the biosynthesis of anthocyanins. For example, mdm-miR828 inhibits anthocyanin accumulation in response to high temperature in apple [77], and miR156 regulates anthocyanin biosynthesis by targeting *SQUAMOSA PROMOTER BINDING PROTEIN-LIKE* (*SPL*) in poplar and *Arabidopsis* [78,79,80]. Moreover, phosphorylated MYB75 is essential for light-induced Arabidopsis anthocyanin accumulation [13].

## 5. Conclusions and Perspectives

Anthocyanins protect against abiotic and biotic stresses in plants, and optimising anthocyanin content is regarded as the goal of breeding programmes in apples and pears. Many factors, such as light, transcription factors, phytohormones, affect the anthocyanin accumulation in plant tissues. Although HY5 and the MBW complex play core roles in anthocyanin biosynthesis in apples, pears and *A. thaliana*, there are still obvious differences in the regulatory mechanism of anthocyanin biosynthesis between different plants. For example, BBX proteins are a link between light signals and HY5 in apples and pears, but PHYTOCHROMES B (PHYB) functions as a “bridge” connecting light stimulus and HY5 in *A. thaliana*. Therefore, it is necessary to elucidate the regulatory mechanisms of anthocyanin biosynthesis in different plant species.

In addition, as follows, there are other problems that also remain to be studied: (1) although the biosynthesis and accumulation of anthocyanins are reportedly regulated by environmental factors, plant hormones, structural genes and regulatory genes, their interrelationships in this pathway need to be further explored. (2) What kind of transcription factor modifications exist in the dynamic balance of anthocyanin synthesis and degradation? Four dominant anthocyanin forms are reversibly transformed at different pH states and affect the colour of aqueous solution. However, what is the mechanism by which pH affects anthocyanin transformation? (3) Current research is limited to the molecular level, but as science and technology progresses, we can strengthen integrated genomic, phenotypic genomics, proteomic, and metabolomic research and use a variety of means to explore unknown parts of anthocyanin regulatory mechanisms and metabolic pathways in apple and pear, and then reveal relationships among anthocyanin and colour, sugar content, and other flavour attributes. In addition, to put this information in perspective, the mechanisms involved in the function of the MBW complex are highly conserved during the regulation of anthocyanin biosynthesis in higher plants, but mechanisms related to environmental and developmental factors affecting anthocyanin biosynthesis are complicated, even the information based on these mechanisms from different species is very fragmented. Thus, additional regulatory pathways and key genes and proteins during the process of anthocyanin biosynthesis in crop, ornamental and other fruit species would also be worthwhile for further investigation.

Overall, with the deepening of research, we will have a clearer understanding of the mechanism of anthocyanin synthesis and accumulation, therefore establishing a stable genetic transformation system using gene engineering technology to improve plant colour, which will provide a solid technology and information basis for crop breeding and mechanised intensive planting in the future.

## Figures and Tables

**Table 1:** Basic information on the six common anthocyanidins.

Name (Abbreviations)	Substitution		Colour	λmax in HCl Acidified MeOH
	R_1_	R_2_		
Pelargonidin (Pg)	H	H	Red	520
Cyanidin (Cy)	OH	H	Magenta	535
Delphinidin (Dp)	OH	OH	Purple	546
Peonidin (Pn)	OCH_3_	H	Magenta	532
Petudinin (Pt)	OCH_3_	OH	Purple	543
Malvidin (Mv)	OCH_3_	OCH_3_	Purple	542
